# Sequencing Anti-CD19 Therapies in Diffuse Large B-Cell Lymphoma: From Mechanistic Insights to Clinical Strategies

**DOI:** 10.3390/ijms26178662

**Published:** 2025-09-05

**Authors:** Filomena Emanuela Laddaga, Mario Della Mura, Joana Sorino, Amanda Caruso, Stefano Martinotti, Giuseppe Ingravallo, Francesco Gaudio

**Affiliations:** 1 Hematology and Cell Therapy Unit, IRCCS Istituto Tumori “Giovanni Paolo II”, 70124 Bari, Italy; 2 Molecular Pathology Section, Department of Precision and Regenerative Medicine and Ionian Area (DiMePRe-J), ‘Aldo Moro’ University, 70124 Bari, Italy; 3 Clinical Pathology Unit, “F. Miulli” University Hospital, 70021 Acquaviva delle Fonti, BA, Italy; 4 Department of Medicine and Surgery, LUM University “Giuseppe Degennaro”, Casamassima, 70010 Bari, Italy; 5 Unit of Hematology, “F. Miulli” University Hospital, 70021 Acquaviva delle Fonti, BA, Italy

**Keywords:** CD19, diffuse large B-cell lymphoma, CAR-T, tafasitamab, loncastuximab, antigen escape, bispecific antibodies, CAR-NK, immunotherapy, treatment sequencing

## Abstract

CD19-targeted therapies, including monoclonal antibodies, antibody–drug conjugates, and chimeric antigen receptor (CAR) T-cell products, have significantly improved outcomes in relapsed/refractory diffuse large B-cell lymphoma (R/R DLBCL). Despite their clinical efficacy, resistance and antigen modulation pose substantial challenges, especially in patients requiring sequential therapy. This review provides a comprehensive overview of CD19 biology and its relevance as a therapeutic target. We examine mechanisms of resistance such as antigen loss, epitope masking, and T-cell exhaustion, as well as the implications of tumor microenvironmental immunosuppression. Future efforts should prioritize the integration of real-time diagnostics, such as flow cytometry, immunohistochemistry, and transcriptomic profiling, and AI-assisted predictive models to optimize therapeutic sequencing and expand access to personalized immunotherapy.

## 1. Introduction

Diffuse large B-cell lymphoma (DLBCL) represents the most prevalent subtype of non-Hodgkin lymphoma and encompasses a clinically and biologically heterogeneous group of aggressive B-cell malignancies [1,2,3,4]. While standard first-line therapy with rituximab plus anthracycline-based chemotherapy (R-CHOP) achieves a cure in a substantial proportion of patients, approximately one-third will develop relapsed or refractory (R/R) disease, for whom prognosis remains poor in the absence of effective salvage strategies [5,6,7].

In recent years, CD19 has emerged as a central immunotherapeutic target in the management of R/R DLBCL. As a pan–B-cell marker expressed throughout B-cell ontogeny and preserved in the majority of B-cell malignancies, CD19 has become the focus of multiple therapeutic platforms, including monoclonal antibodies (e.g., tafasitamab), antibody–drug conjugates (e.g., loncastuximab tesirine), and chimeric antigen receptor T-cell (CAR-T) therapies (e.g., axi-cel, tisa-cel, and liso-cel). Each of these agents engages CD19 through distinct mechanisms of action, offering unique clinical opportunities and challenges for therapeutic sequencing [8,9,10,11,12].

In addition to these established modalities, CD19-targeted bispecific and trispecific antibodies, commonly referred to as BiTEs, have gained increasing relevance in clinical practice. These agents redirect immune effector cells toward malignant B-cells and are now part of the therapeutic arsenal for R/R DLBCL.

However, the optimal positioning of CD19-directed therapies within the treatment continuum remains undefined. Data from pivotal trials are limited by the frequent exclusion of patients previously treated with other CD19-targeted agents, and standardized methods to assess the expression level and persistence of CD19 are lacking. Furthermore, emerging evidence suggests that resistance mechanisms, including antigen loss, alternative splicing, epitope masking, and immune evasion, may differentially impact therapeutic efficacy across drug classes [13,14,15,16].

Understanding the molecular mechanisms underlying CD19 expression and modulation is essential for optimizing the clinical use of CD19-targeted therapies. In particular, insights into antigen density, epitope accessibility, and resistance mechanisms provide a critical foundation for therapeutic decision-making. This review integrates mechanistic knowledge with clinical evidence to explore how biological variability informs the sequencing of tafasitamab, loncastuximab, CAR-T cell therapies, and emerging platforms such as CAR-NK cells and BiTEs. This review aims to provide a comprehensive analysis of CD19 biology and its therapeutic relevance in DLBCL, with a specific focus on mechanisms of resistance, the impact of prior CD19-directed therapy, and the clinical feasibility of sequential targeting. Additionally, we discuss evolving treatment strategies beyond T-cell-based platforms, including CAR-NK cells and trispecific antibodies, and propose future directions for research and clinical integration.

## 2. CD19 as Therapeutic Target

**Figure 1:**
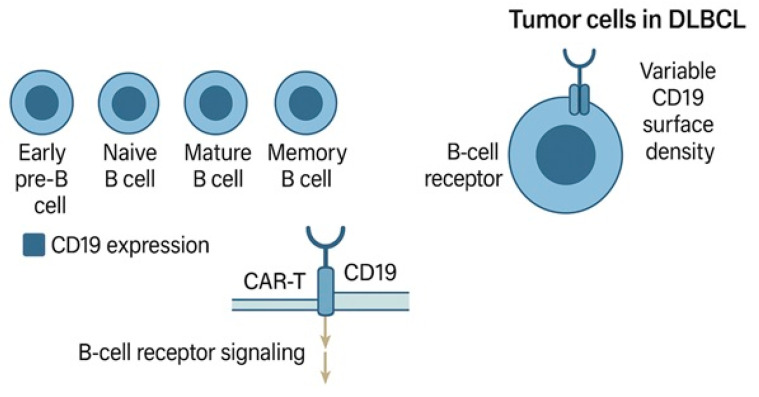
CD19 biology and its therapeutic relevance in DLBCL. CD19 acts as a coreceptor in B-cell signalling and is preserved across most B-cell malignancies, making it an ideal immunotherapeutic target.

CD19 is a transmembrane glycoprotein of the immunoglobulin superfamily expressed almost ubiquitously throughout B-cell development, from the early pre-B-cell stage through mature memory B-cells. Its expression is lost only upon terminal differentiation into plasma cells. Functionally, CD19 acts as a coreceptor that amplifies B-cell receptor (BCR) signalling, lowers the threshold for antigen stimulation, and contributes to the regulation of B-cell activation and survival [17,18,19,20,21]. However, in contrast to other potential targets, CD19 is not considered an oncogenic driver in DLBCL, and its role is predominantly as a lineage-specific marker rather than a functional dependency (Figure 1).

This unique biological profile has rendered CD19 an attractive immunotherapeutic target, with several key advantages. First, CD19 expression is preserved in the vast majority of B-cell malignancies, including nearly all cases of DLBCL at diagnosis. Second, its lack of expression in hematopoietic stem cells and non-B-cell lineages minimizes the risk of off-tumor toxicity. Third, its internalization properties, though modest, are sufficient to enable the delivery of cytotoxic payloads via antibody–drug conjugates [20,21].

Nonetheless, the mere presence of CD19 does not guarantee therapeutic efficacy. Increasing evidence suggests that CD19 antigen density, defined as the number of CD19 molecules expressed on the tumor cell surface, may influence the activity of CD19-targeted agents, particularly CAR-T cells. Preclinical models have demonstrated a correlation between target antigen density and CAR-T–mediated cytotoxicity, with CD19-CD28ζ constructs showing greater sensitivity to low-density targets than CD19–4-1BBζ-based products [21].

Detection of CD19 expression in clinical practice is typically performed using immunohistochemistry (IHC) or flow cytometry. IHC offers wide applicability on formalin-fixed paraffin-embedded tissues, but suffers from limited dynamic range and potential inter-observer variability. Flow cytometry, when applicable, allows for quantitative assessment and detection of low-abundance antigens, yet is restricted to liquid samples such as peripheral blood or bone marrow aspirates. Neither technique has been prospectively validated for guiding therapeutic decisions based on CD19 antigen density thresholds [22].

Furthermore, CD19 expression is not static and may evolve over the course of therapy. Post-treatment changes in expression may arise from several mechanisms, including genetic mutations (e.g., in exons 2–4), alternative mRNA splicing leading to truncated isoforms, transcriptional downregulation, or even epitope masking due to antibody binding. These changes can be either permanent or transient and may vary depending on the specific CD19-targeted agent administered, the duration of exposure, and the interval between treatments [23].

Notably, emerging clinical data suggest that CD19 may remain detectable after exposure to certain therapies, particularly monoclonal antibodies such as tafasitamab, albeit at reduced density or with altered conformational accessibility. This finding has critical implications for the feasibility of sequential CD19-directed therapies, which increasingly require precise, time-sensitive, and functionally relevant assessments of antigen status [24].

Different CD19-targeted therapies exert distinct effects on antigen expression, which have important implications for subsequent treatment strategies. Tafasitamab, a monoclonal antibody, is primarily associated with epitope masking without significant internalization, thereby preserving CD19 availability for future targeting. In contrast, loncastuximab tesirine, an antibody–drug conjugate, induces internalization and degradation of CD19 through its cytotoxic payload. CAR-T cell therapies, which rely on robust CD19 expression for activation, are more frequently associated with antigen loss due to genetic mutations, alternative splicing, or transcriptional downregulation. These mechanistic differences influence the feasibility and timing of sequential CD19-directed therapies. A comparative overview of these effects is provided in Table 1, which summarizes the mechanisms of action, clinical efficacy, and implications for re-targeting across the main CD19-directed platforms.

## 3. CD19-Directed Therapies in Relapsed/Refractory DLBCL

The therapeutic landscape of R/R DLBCL has undergone a paradigm shift with the introduction of agents specifically targeting CD19 (Table 2). These therapies, though united by a common antigenic target, differ significantly in their mechanisms of action, clinical indications, safety profiles, and implications for subsequent treatment sequencing. Three main classes currently dominate the field: monoclonal antibodies, antibody–drug conjugates, and autologous CAR-T cell therapies [25,26,27].

### 3.1. Tafasitamab in Combination with Lenalidomide

Tafasitamab is a humanized, Fc-enhanced monoclonal antibody targeting CD19, engineered to augment antibody-dependent cellular cytotoxicity (ADCC) and phagocytosis. In combination with lenalidomide, it received accelerated approval based on the phase II L-MIND trial, which enrolled transplant-ineligible patients with R/R DLBCL and ≤2 prior lines of therapy [28]. The combination demonstrated an objective response rate (ORR) of 57.5%, with a complete response (CR) rate of 40% and a median duration of response exceeding 43 months in updated analyses [29,30,31].

Subgroup analyses from the L-MIND study have identified specific clinical characteristics associated with improved outcomes following tafasitamab plus lenalidomide treatment. Patients with non-bulky disease, low International Prognostic Index (IPI) scores, and preserved immune function—particularly natural killer (NK) cell activity—demonstrated higher response rates and longer durations of remission. Additionally, those with ≤2 prior lines of therapy and good performance status (ECOG 0–1) were more likely to benefit from this combination. These findings support the use of tafasitamab in biologically and clinically favorable subsets of relapsed/refractory DLBCL.

The therapeutic synergy between tafasitamab and lenalidomide is believed to stem from the latter’s immunomodulatory effects, including enhancement of natural killer (NK) cell–mediated cytotoxicity. Notably, tafasitamab does not induce rapid CD19 internalization, thereby preserving antigen availability for potential subsequent CD19-targeted therapies [8,32].

However, real-world evidence suggests a degree of heterogeneity in response durability, particularly in patients with high-risk cytogenetics or bulky disease. Moreover, the immunologic effects of lenalidomide may be blunted in heavily pretreated or immunocompromised patients, potentially limiting efficacy in later lines of therapy.

### 3.2. Loncastuximab Tesirine

Loncastuximab tesirine is an antibody–drug conjugate (ADC) comprising a humanized anti-CD19 antibody linked to a pyrrolobenzodiazepine (PBD) dimer toxin via a protease-cleavable linker. It was approved based on the phase II LOTIS-2 trial, in which it demonstrated an ORR of 48.3% and a CR rate of 24.1% in a heavily pretreated cohort (median of 3 prior therapies) [33,34,35,36].

In the LOTIS-2 trial, loncastuximab tesirine showed variable efficacy across patient subgroups. Patients with fewer prior lines of therapy (≤3), absence of significant hepatic comorbidities, and lower tumor burden achieved higher objective response rates. Moreover, those with preserved bone marrow function and adequate platelet counts tolerated treatment better and experienced fewer dose-limiting toxicities. These observations suggest that loncastuximab may be particularly suitable for patients with intermediate-risk disease and limited prior exposure to cytotoxic agents.

Unlike tafasitamab, loncastuximab exerts direct cytotoxic effects via internalization of the antibody–toxin complex and subsequent DNA cross-linking. The pharmacodynamic requirement for CD19 internalization may influence its efficacy in tumors with variable expression or epitope accessibility [37].

The toxicity profile of loncastuximab includes thrombocytopenia, edema, and liver enzyme elevations, which may limit its use in frail or comorbid patients. Importantly, its use post-CAR-T therapy is currently permitted, although real-world data on sequencing remain sparse [38].

### 3.3. Anti-CD19 CAR-T Cell Therapies

CAR-T therapies targeting CD19, namely axicabtagene ciloleucel (axi-cel), tisagenlecleucel (tisa-cel), and lisocabtagene maraleucel (liso-cel), have transformed the management of R/R DLBCL in fit patients. These autologous cell products differ in costimulatory domains, manufacturing platforms, and toxicity profiles, yet all share a reliance on robust CD19 expression for effective target engagement [39]. Axi-cel (CD28 costimulation) demonstrated superior progression-free survival compared to standard of care in the ZUMA-7 trial for second-line treatment, though it carries a higher risk of cytokine release syndrome (CRS) and neurotoxicity [40]. Tisa-cel (4-1BB costimulation) offers a more favorable toxicity profile but with slower expansion kinetics, potentially impacting efficacy in patients with aggressive disease kinetics 41. Liso-cel combines 4-1BB costimulation with a defined composition of CD4/CD8+ cells and has demonstrated favorable efficacy and tolerability in both the third-line and second-line settings [41]. While CD19 expression is almost universally present at baseline, antigen modulation or loss has been observed in patients relapsing after CAR-T therapy. Furthermore, manufacturing times, logistical complexity, and lymphodepleting chemotherapy requirements limit accessibility and feasibility in certain clinical contexts.

Collectively, these agents provide complementary approaches to targeting CD19, each with distinct advantages and limitations. Their expanding use across treatment lines necessitates a careful understanding of cross-resistance mechanisms, timing of administration, and potential implications for sequential CD19 re-targeting.

## 4. Dynamics of CD19 Expression

**Figure 2:**
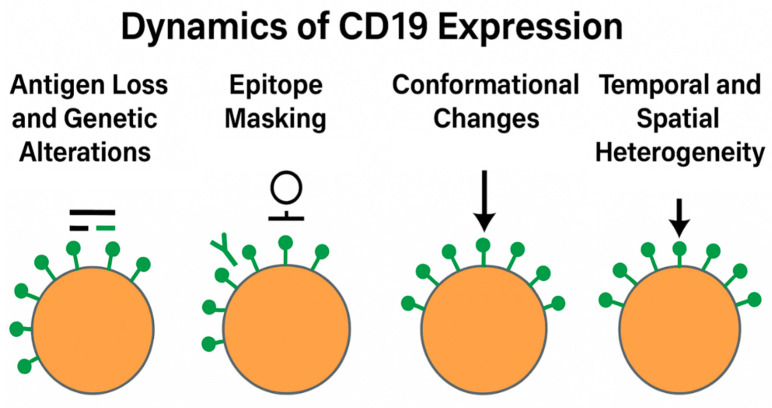
Dynamics of CD19 expression. CD19 modulation can occur via genetic mutations, epitope masking, and transcriptional changes, influencing the feasibility of sequential therapies.

Although CD19 is almost universally expressed in treatment-naïve DLBCL, its expression is not immutable. Growing evidence suggests that CD19 surface availability may evolve during the disease course, particularly in response to selective pressure from immunotherapeutic agents. Understanding the molecular and phenotypic dynamics of CD19 expression is therefore critical for informing therapeutic sequencing and anticipating resistance mechanisms (Figure 2).

### 4.1. Antigen Loss and Genetic Alterations

Antigen escape represents a major mechanism of resistance to CD19-directed therapies [42]. This phenomenon encompasses both quantitative and qualitative changes in CD19 expression, including:

- Genetic mutations, such as insertions, deletions, and point mutations in the CD19 gene, particularly affecting exon 2, which encodes the scFv-binding region of CAR constructs;
- Alternative splicing events, generating truncated isoforms lacking extracellular epitopes, as initially reported in pediatric acute lymphoblastic leukemia and more recently observed in high-grade B-cell lymphomas;
- Transcriptional downregulation or epigenetic silencing, such as promoter hypermethylation, although the latter appears to be a less prevalent mechanism in DLBCL.

Importantly, these alterations may render malignant cells invisible to CD19-directed immune effectors, including CAR-T cells and monoclonal antibodies [17,43,44].

Emerging preclinical studies have investigated strategies to reverse CD19 downregulation and restore antigen expression following therapeutic pressure. Epigenetic modulation, such as the use of DNA demethylating agents, has shown potential in reactivating silenced CD19 transcription. Cytokine stimulation—particularly with interleukin-15 (IL-15)—may enhance CD19 surface expression and immune recognition. Additionally, gene editing technologies, including CRISPR/Cas9-based approaches, are being explored to correct CD19 mutations or restore functional isoforms. While these interventions remain experimental, they offer promising avenues to overcome resistance and expand the feasibility of CD19 re-targeting in relapsed/refractory B-cell malignancies.

### 4.2. Epitope Masking and Conformational Changes

In some cases, CD19 is retained at the transcript and protein level but becomes functionally inaccessible due to:

- Epitope masking, wherein prior therapeutic antibodies occupy key binding sites required for subsequent agents (e.g., tafasitamab pre-treatment, impairing CAR-T binding);
- Steric hindrance or receptor internalization induced by antibody engagement;
- Altered glycosylation patterns, which can modulate antigen structure and impair immune recognition.

These mechanisms may lead to false-negative CD19 assessments and underestimate the feasibility of sequential targeting [15,45,46].

### 4.3. Temporal and Spatial Heterogeneity

The dynamics of CD19 expression are also influenced by:

Temporal variation, whereby antigen modulation is transient and may recover after a sufficient washout period;

Spatial heterogeneity, with subclonal populations exhibiting differential CD19 density or isoform composition.

Such heterogeneity underscores the limitations of single-timepoint assessments and supports the use of serial monitoring by flow cytometry, immunohistochemistry (IHC), or transcriptomic profiling to inform treatment decisions [47].

### 4.4. Implications for Sequential Therapy

Clinical studies have demonstrated that CD19 expression is frequently preserved at relapse following CD19-targeted therapy, although density and accessibility may be altered. For instance, Duell et al. [46] showed that patients relapsing after tafasitamab retained detectable CD19 by IHC and mRNA analysis, supporting the rationale for subsequent CD19-targeting, including CAR-T therapy, provided that epitope masking is resolved [45,48].

Conversely, CD19-negative relapses following CAR-T cell therapy, particularly axi-cel, have been associated with deleterious CD19 mutations or complete antigen loss, potentially limiting the utility of further CD19-directed approaches [49,50].

The dynamic nature of CD19 thus represents both a challenge and an opportunity: while it complicates therapeutic planning, it also suggests that resistance may be context-dependent and reversible, allowing for rational re-engagement of the same target under appropriate conditions [51].

## 5. Sequential Use of CD19 Therapies

The increasing availability of distinct CD19-directed immunotherapeutics has raised the question of whether sequential targeting of the same antigen is feasible and under which biological and clinical circumstances it might be effective. Although historically the concern for CD19 antigen loss or masking led to caution in reusing this target, both preclinical models and real-world data are beginning to support a more nuanced view [48,52,53].

The feasibility of sequential CD19-targeted therapy is closely linked to the persistence of CD19 expression following prior treatment. Available data suggest that CD19 is frequently retained after exposure to monoclonal antibodies such as tafasitamab, albeit with reduced density or altered epitope accessibility. For example, Duell et al. [46] demonstrated that CD19 remained detectable by IHC and mRNA analysis in patients relapsing after tafasitamab, supporting the rationale for subsequent CD19-directed therapies, including CAR-T cells.

Conversely, CD19 loss is more commonly observed following CAR-T therapy, particularly axi-cel, due to mechanisms such as genetic mutations, alternative splicing, or transcriptional silencing. Epperla et al. [52] reported that CD19-negative relapses post-CAR-T were associated with poor responses to further CD19-targeted agents.

While these findings highlight the importance of reassessing antigen status before re-challenging the CD19 axis, it is important to note that large-scale prospective data comparing outcomes in CD19^+^ vs CD19^−^ patients remain limited. Future studies should prioritize stratification by antigen status to better define the efficacy of sequential CD19 therapies.

### 5.1. Preclinical and Real-World Evidence

Preclinical studies have demonstrated that the CD19 antigen may persist after exposure to certain anti-CD19 agents, particularly monoclonal antibodies. In murine models, sequential administration of tafasitamab followed by CD19 CAR-T cells, after a defined washout period, preserved CAR-T expansion and cytotoxicity, and was associated with attenuated CRS compared to concurrent use. These findings suggest that the immunologic impact of prior anti-CD19 exposure may be modulated by timing, dosing, and mechanism of action [22,26,54,55].

Real-world data, although still limited, echo these observations. Duell et al. [46] reported that patients relapsing after tafasitamab retained detectable CD19 expression by IHC and mRNA analysis, implying epitope masking rather than complete antigen loss [52,56].

### 5.2. Post-CAR-T CD19 Re-Targeting

Reutilization of CD19-directed therapy after CAR-T cell failure is more controversial. In the LOTIS-2 trial, patients receiving loncastuximab tesirine after prior CAR-T therapy achieved an ORR of approximately 36%, suggesting retained sensitivity in a subset of cases. Notably, these outcomes may reflect both preserved CD19 expression and alternative cytotoxic mechanisms independent of T-cell fitness [57,58].

Similarly, anecdotal reports and retrospective analyses have documented responses to tafasitamab-based regimens post-CAR-T, albeit with reduced efficacy in patients exhibiting evidence of CD19-negative relapse or profound immunologic exhaustion. These findings reinforce the need for antigen reassessment prior to re-challenging the CD19 axis, using multimodal techniques such as flow cytometry, IHC, and molecular profiling.

### 5.3. The Role of Epitope Accessibility and Washout

A critical, yet often underappreciated, factor in sequential CD19 targeting is epitope availability. Tafasitamab and most CAR constructs bind overlapping extracellular domains of CD19; if administered in close temporal proximity, residual antibody binding may sterically hinder CAR-T cell recognition. Consequently, a washout period of at least 2–3 weeks, supported by both in vitro and clinical data, has been proposed to allow dissociation of previously bound antibodies and restoration of functional CD19 epitopes [45,59].

This concept of epitope unmasking has direct implications for clinical practice. In patients who progress shortly after receiving tafasitamab or loncastuximab, early re-targeting with CAR-T or bispecific antibodies may be less effective unless adequate time is allowed for epitope recovery. Conversely, a prolonged interval between CD19-directed therapies may increase the likelihood of successful re-engagement [15,60].

## 6. Alternative Approaches Beyond T-Cells

While CD19-directed CAR-T therapy has demonstrated transformative potential in R/R DLBCL, its widespread application is limited by factors including toxicity, manufacturing delays, and the requirement for adequate T-cell fitness. As a result, considerable effort has been directed toward developing non-T-cell-based platforms that retain CD19 specificity while offering distinct pharmacologic, logistical, and immunologic advantages [61,62,63].

### 6.1. CD19-Directed CAR-NK Cells

CAR-engineered natural killer (CAR-NK) cells represent a novel class of cell-based immunotherapy with several theoretical and practical benefits over CAR-T cells. Unlike autologous CAR-T products, CAR-NK therapies can be derived from allogeneic sources—including cord blood, peripheral blood, or induced pluripotent stem cells (iPSCs)—and manufactured as off-the-shelf products, thus overcoming delays associated with patient-specific engineering [64,65,66,67].

From an immunologic standpoint, CAR-NK cells exhibit:

- A lower risk of CRS and immune effector cell-associated neurotoxicity syndrome (ICANS),
- Intrinsic cytotoxicity independent of CAR activation, enabling recognition of CD19-low or CD19-negative targets via natural killer receptors,
- Minimal risk of graft-versus-host disease (GVHD), enabling use in heavily pretreated or immunocompromised populations.

In a landmark phase I trial by Liu et al., cord blood–derived anti-CD19 CAR-NK cells administered to patients with lymphoid malignancies demonstrated a favorable safety profile with encouraging efficacy signals and no CRS or neurotoxicity 68. More recently, early-phase studies have explored iPSC-derived CAR-NK constructs, such as FT596, in aggressive B-cell lymphomas, with preliminary data confirming feasibility and early clinical activity.

Despite their promise, CAR-NK platforms remain investigational, and their durability, scalability, and clinical efficacy in comparison to CAR-T therapies require validation in randomized trials [68,69,70,71].

### 6.2. Bispecific and Trispecific CD19-Directed Antibodies

**Figure 3:**
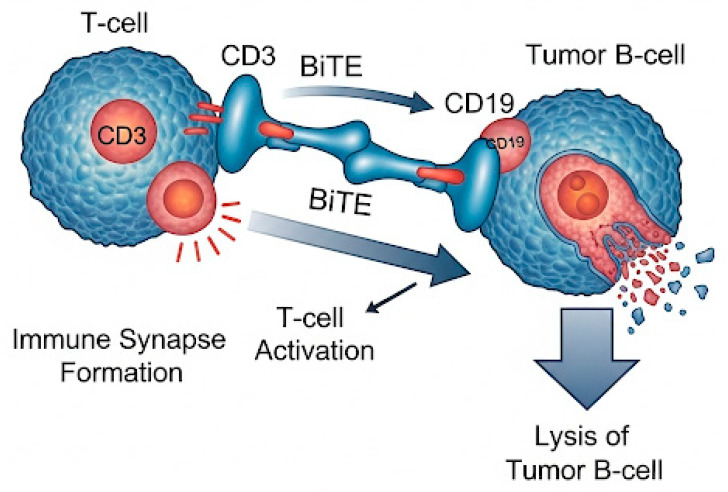
Mechanism of action of CD19-directed BiTEs (Bispecific T-cell Engagers). This figure illustrates the immunologic mechanism by which CD19-directed BiTEs mediate tumor cell killing in diffuse large B-cell lymphoma (DLBCL). The BiTE molecule simultaneously binds to CD3 on the surface of a T-cell and CD19 on a malignant B-cell, facilitating immune synapse formation and T-cell activation, ultimately leading to tumor cell lysis.Dynamics of CD19 expression. CD19 modulation can occur via genetic mutations, epitope masking, and transcriptional changes, influencing the feasibility of sequential therapies.

Bispecific T-cell (Figure 3) engagers (BiTEs) and trispecific antibodies targeting CD19 constitute another frontier in immunotherapy, offering off-the-shelf, non-cellular strategies for redirecting immune effector cells toward malignant B-cells.

Bispecific antibodies simultaneously bind CD3 on T-cells and CD19 on B-cells, promoting T-cell activation, immunologic synapse formation, and target cell lysis. Unlike CAR-T cells, these agents are not reliant on patient-derived lymphocytes and are suitable for rapid administration, including in the outpatient setting [72,73,74,75].

Recent developments have led to the engineering of trispecific constructs that engage multiple tumor-associated antigens (e.g., CD19 and CD20) and/or costimulatory receptors (e.g., CD28 or CD2), enhancing cytotoxic potency and potentially mitigating antigen escape. These next-generation molecules aim to:

- Reduce the risk of immune evasion through multi-antigen targeting,
- Promote more robust and sustained T-cell activation,
- Maintain therapeutic activity in the setting of prior CAR-T failure or antigen heterogeneity.

While clinical data remain early, bispecific and trispecific antibodies are poised to play an important role in treatment paradigms where T-cell-based therapies are infeasible, contraindicated, or have failed. Alternative CD19-directed strategies, particularly CAR-NK cells and multi-specific antibodies, offer promising avenues to expand therapeutic access, reduce toxicity, and overcome resistance mechanisms inherent to T-cell-based platforms. Their integration into future treatment algorithms will depend on maturing clinical evidence, logistical feasibility, and biomarker-driven patient selection [72,76,77].

As bispecific and trispecific CD19-directed antibodies continue to gain traction in clinical settings, their integration into treatment algorithms warrants careful consideration. These agents offer off-the-shelf accessibility, rapid immune activation, and multi-antigen targeting capabilities, making them particularly valuable in patients who are ineligible for cellular therapies or have relapsed after CAR-T treatment. Future studies should explore their optimal sequencing alongside other CD19-targeted modalities, and predictive biomarkers may help guide their use in specific patient subgroups.

## 7. Conclusions and Future Perspectives

The emergence of CD19-directed immunotherapeutics has redefined the management landscape of R/R DLBCL, offering new opportunities for durable remissions in patients with limited options. However, the expanding number of available agents and the complexity introduced by resistance, antigen dynamics, and immune dysfunction have underscored the need for a more personalized and biologically informed approach to therapy selection and sequencing. To this end, future treatment algorithms must integrate predictive models that incorporate multiple layers of clinical and biological data, including:

- CD19 antigen status, encompassing expression level, isoform integrity, and epitope accessibility;
- Tumor microenvironment (TME) characteristics, such as immunosuppressive infiltrates and cytokine profiles;
- Treatment history, particularly prior exposure to CD19-directed agents and their immunologic sequelae;
- Host-related factors, including immune competence and eligibility for cellular therapy.

Central to this strategy is the dynamic assessment of CD19, which requires the integration of modern diagnostic platforms beyond baseline IHC. Techniques such as serial flow cytometry, RNA sequencing, and digital spatial profiling can provide high-resolution, time-sensitive insights into antigen persistence and immune contexture, enabling real-time therapeutic decision-making.

Ultimately, the development of AI-driven predictive platforms, capable of integrating molecular profiling, immune signatures, radiologic features, and real-world treatment trajectories, holds promise for optimizing therapeutic sequencing. Prospective, biomarker-informed trials and real-world registries should aim to validate these tools and guide their clinical implementation. As our understanding of CD19 dynamics deepens, the future of R/R DLBCL treatment lies not only in targeting the right antigen, but in targeting it at the right time, in the right context, and with the right platform.

## Figures and Tables

**Table 1:** Comparative overview of CD19-targeted therapies, their mechanisms of action, effects on CD19 expression, and implications for sequential treatment strategies.

Therapy	Mechanism of Action	Effect on CD19 Expression	Implications for Sequential Therapy
Tafasitamab	Fc-enhanced monoclonal antibody inducing ADCC and phagocytosis	Epitope masking without significant internalization	CD19 often preserved; suitable for re-targeting
Loncastuximab Tesirine	Antibody–drug conjugate with internalization and DNA cross-linking	Internalization and degradation of CD19	Reduced antigen density may affect subsequent targeting
CAR-T Cell Therapies	Autologous T-cells engineered to target CD19	Antigen loss via mutation, splicing, or downregulation	Loss of CD19 may preclude further CD19-directed therapy

**Table 2:** Overview of CD19-directed therapies in R/R DLBCL.

Therapy	ORR (%)	CR Rate (%)	Median OS (Months)
Tafasitamab + Lenalidomide	57.5	40	Not reached
Loncastuximab Tesirine	48.3	24.1	9.5
Axi-cel	83	65	24.4
Tisa-cel	52	39	11.1
Liso-cel	73	53	18.1
CD19 BiTEs	43	19	10
